# Cognitive Functioning of Glucocerebrosidase (*GBA*) Non-manifesting Carriers

**DOI:** 10.3389/fneur.2021.635958

**Published:** 2021-02-26

**Authors:** Eileen E. Moran, Susan B. Bressman, Roberto A. Ortega, Deborah Raymond, William C. Nichols, Christina A. Palmese, Sonya Elango, Matthew Swan, Vicki Shanker, Imali Perera, Cuiling Wang, Molly E. Zimmerman, Rachel Saunders-Pullman

**Affiliations:** ^1^Department of Psychology, Fordham University, Bronx, NY, United States; ^2^Department of Psychiatry, Massachusetts General Hospital, Boston, MA, United States; ^3^Department of Neurology, Mount Sinai Beth Israel Medical Center, New York, NY, United States; ^4^Department of Neurology, Ichan School of Medicine at Mount Sinai, New York, NY, United States; ^5^Division of Human Genetics, Department of Pediatrics, Cincinnati Children's Hospital Medical Center, University of Cincinnati College of Medicine, Cincinnati, OH, United States; ^6^Department of Epidemiology and Family Health, Albert Einstein College of Medicine, Yeshiva University, Bronx, NY, United States

**Keywords:** glucocerebrosidase, GBA, cognition, executive functioning, Parkinson's disease

## Abstract

Mutations and variants in the glucocerebrosidase (*GBA*) gene are among the most common genetic risk factors for the development of Parkinson's disease (PD). Yet, penetrance is markedly reduced, and less is known about the burden of carrying a single mutation among those without diagnosed PD. Motor, cognitive, psychiatric, and olfactory functioning were assessed in 30 heterozygous *GBA* mutation carriers without PD (the majority of whom had mild *GBA* mutations) and 49 non-carriers without PD. Study focus was on domains affected in *GBA* mutation carriers with PD, as well as those previously shown to be abnormal in *GBA* mutation carriers without PD. *GBA* mutation carriers showed poorer performance on the Stroop interference measure of executive functioning when controlling for age. There were no group differences in verbal memory, Montreal Cognitive Assessment (MoCA), overall motor score, or presence of REM sleep behavior disorder or depression. Although total olfaction scores did not differ, *GBA* mutation carriers with hyposmia had lower global cognition scores than those without hyposmia. As anticipated by the low penetrance of *GBA* mutations, these findings suggest that pre-manifest non-motor or motor features of PD may not present in most *GBA* mutation carriers. However, there is support that there may be a subtle difference in executive functioning among some non-manifesting heterozygous *GBA* mutation carriers, and, combined with olfaction, this may warrant additional scrutiny as a potential biomarker for pre-manifest and pre-clinical *GBA* related PD.

## Introduction

*GBA* mutations are a common genetic risk factor for Parkinson disease (PD) and dementia with Lewy Bodies (DLB) (1–3). While harboring two copies of certain *GBA* mutations may lead to Gaucher disease, both mono- and biallelic *GBA* mutation carriers are at an increased risk of developing PD. *GBA* related PD (*GBA*-PD) may have earlier age of onset and more prominent non-motor features than idiopathic PD (4–6). This includes an increased risk of mild cognitive impairment (7) and dementia (8–10). *GBA*-PD may exhibit a specific cognitive profile, with greater weakness in working memory/executive functioning (9), and visuospatial processing relative to idiopathic PD (9). Further, some studies have indicated more significant symptoms of depression, apathy, anxiety, and REM sleep behavior disorder (RBD) overall, or in a subset in *GBA*-PD (4, 11). Biallelic carriers may have significant olfactory disturbance (12), and monoallelic carriers experience more olfactory dysfunction relative to non-carriers (4), though the relationship between *GBA* status and olfaction has not been universally demonstrated (13).

Although earlier age at onset (14), and greater burden of both motor decline (15) and non-motor symptoms (16) are more pronounced among carriers of more “severe” GBA mutations, this phenotype is also reported among carriers of more “mild” mutations (16, 17). However, among carriers of mild GBA mutations, this prominent non-motor phenotype may manifest later in the disease course. A recent large scale, multi-center study suggests that individuals with *GBA*-PD, who are carriers of a mild *GBA* mutation (N409S), displayed a PD phenotype that was similar to non-mutation, sporadic PD, during the first 3 years of clinical disease (13).

Penetrance of *GBA* mutations for the development of PD is markedly reduced. A recent large scale investigation estimates that for monoallelic carriers, the risk of developing PD was 10% at age 60, 16% at age 70, and 19% by age 80 (18). Prior penetrance estimates assessing cohorts with known family history of PD report higher penetrance, reflecting either ascertainment bias or shared additional genetic factors (18). This overall risk further varies in relation to mutation severity (19). Severe mutations confer a 13.6-fold increased risk, while mild mutations confer a 2.2-fold increased risk for the development of PD (20, 21). Less is known about the burden of harboring a single *GBA* mutation outside of the context of PD. With emerging investigations of therapeutics targeting underlying disease process associated with *GBA* mutations (14), detailed characterization of *GBA* carriers is essential in order to determine early, pre-clinical markers of phenoconversion.

Recent studies have examined the prodromal course prior to PD onset, as well as other *GBA* associated conditions, such as DLB and RBD. However, findings vary in the extent of cognitive involvement in non-manifesting *GBA* mutation carriers (*GBA*-NMC), which may be attributable to mutation type and ascertainment. Studies investigating cohorts of mild or mild and severe mutation carriers ascertained in clinical settings and thus often consisting of relatives of individuals with PD, (15, 22, 23) have reported differences in global cognitive functioning, frequently assessed using the Montreal Cognitive Assessment (MoCA). In one study, *GBA*-NMC did not show worsening of MoCA score over a 6 year follow up period (23). We previously reported data from a community-based study in which carriers of mild mutations exhibited subtle decline in verbal memory (24).

In this study, we aim to extend prior work, assessing cognitive and other features among *GBA*-NMC relative to peers, in a sample comprised of first-degree relatives, spouses, and friends of PD patients from our outpatient clinic. This sample straddles the community based sample and a purely clinic based sample, and might be more representative of the cross-section of individuals in a New York sample who may seek counseling for *GBA* mutations.

## Materials and Methods

### Participants

Participants were recruited as part of a larger ongoing study assessing the genetics of PD at Mount Sinai Beth Israel. Participants included in this analysis did not have PD. They participated because they had a first or second degree relative with PD, were spouses of individuals with known PD, or were community volunteers without family history of PD. Participants were evaluated by a movement disorders specialist neurologist, and a diagnostic checklist was completed that evaluated presence of clinical symptoms of a movement disorder. Participants with PD, cognitive impairment, or other major neurological diseases were excluded, as were those who also harbored a G2019S *LRRK2* mutation or Gaucher disease.

Inclusion as either a non-manifesting *GBA* carrier (NMC) or mutation negative control was based on the results of genotyping, and some spouses of PD patients harbored *GBA* mutations. *GBA* mutation/variant status was determined as previously described (12, 25). In brief, participants were screened for the eight most common *GBA* mutations (N409S, L483P, 84GG, IVS2+1, V433L, del55bp, D448H, and R535H) as well as E365K, T408M, and the G2019S *LRRK2* mutation. The Tag-It™ Mutation Detection Kit (Luminex Molecular Diagnostics, Toronto, ON, Canada) was used to perform genotyping according to the manufacturer's instructions. Using this system, the regions around the target genes were amplified by multiplex polymerase chain reaction (PCR). The regions were subjected to allele-specific primer extension, hybridized to specific Luminex® beads via Universal Tags, and sorted on a Luminex® 100 IS platform (Luminex Corporation, Austin, TX, USA). Genotyping was then completed using the Tag-It™Data Analysis Software (Luminex Molecular Diagnostics).

Participants provided written informed consent, and this study protocol was approved by the Mount Sinai Institutional Review Board.

### Measures

Systematic neurological history and examination, including Unified Parkinson Disease Rating Scale-III (UPDRS-III), was completed by a neurologist. Non-motor symptoms were assessed using neuropsychological measures, administered by trained coordinators under the guidance of a clinical neuropsychologist. The following cognitive measures were used: Montreal Cognitive Assessment (MoCA), Hopkins Verbal Learning Test—Revised (HVLT-R), Stroop Test, Color Trails Test, Symbol Digit Modality Test, Digit Span, Letter Number Sequencing, Judgment of Line Orientation, FAS, and Animal Fluency.

Additional non-motor features were assessed using the Beck Depression Inventory—II (BDI-II), State-Trait Anxiety Inventory (STAI), REM Sleep Behavior Disorder Questionnaire (RBDQ), and University of Pennsylvania Smell Identification Test (UPSIT).

### Analysis

As prior assessments have found differences between *GBA*-NMC and controls in motor functioning (15), both specific cognitive domains (24, 26) and overall cognition, depression, RBD, and olfaction (15), our primary hypothesis-driven analyses were on the effect of specific *GBA* status in these domains. In particular, primary cognitive analyses were performed on assessment of verbal memory (HVLT-R), executive function (Stroop), and MoCA. Primary motor comparisons were associated with continuous UPDRS-III scores. Depression (BDI-II) and RBD (RBDQ) were assessed using recommended cut scores of 14 and 5, respectively. As there are two major methods to assess olfaction, olfactory performance on the UPSIT was compared both continuously and dichotomously, using a cut-off score of 15 percentile adjusted for age and gender to define hyposmia (27).

Summaries of baseline demographics and outcome measures were presented and compared using parametric or nonparametric tests as appropriate (Table 1). Cognitive measures are reported in demographically adjusted standardized scores. For all primary outcomes, linear regression models were performed to assess the effect of mutation status on motor and non-motor functioning adjusting for age and sex when indicated by significant associations between outcome and demographics. Exploratory analyses evaluated additional cognitive domains (attention, working memory, processing speed, verbal learning, verbal fluency, and visuospatial functioning) and anxiety, as well as the relationships between olfaction and other domains. All analyses were conducted using SPSS 25 (SPSS INC., Chicago, IL, USA).

**Table 1:** Univariate summary of demographics and outcome variables.

	**Total (*N* = 79)**	***GBA*-NMC (*n* = 30)**	**Non-carrier controls (*n* = 49)**
**Demographics**			
Age, years, *mean (SD), range*	57.63 (12.53), 22–89	53.93 (14.41), 29–89	59.90 (12.58), 22–86
*Median (IQR)*	60.00 (48.00–66.00)	59.50 (38.75–63.25)	61.00 (52.50–68.50)
Education, *mean (SD)*	17.00 (2.00), 12–20	17.13 (1.78), 12–20	16.78 (6.23), 12–20
Gender, *n(%) Female*	23 (29.1%)	11 (36.7%)	12 (24.5%)
Family history of PD, *n(%)*	24 (30.4%)	17 (56.7%)	7(14.3%)
**Primary analyses**			
UPDRS-III, *mean (SD), range*	0.72 (1.29), 0.0–5.0	0.75 (1.19), 0.0–5	0.70 (1.36), 0.0–5.0
MoCA total score, *mean (SD), range*	27.72 (1.89), 21–30	27.60 (2.06), 23–30	27.78 (1.81), 21–30
HVLT-R Recall, *z* score, *mean (SD), range*	−0.31 (1.21), −3.22–1.22	−0.36 (0.85), −1.94 to 1.00	−0.27 (1.42), −3.22 to 1.22
Stroop Interference, *T*-score, *mean (SD), range*	49.00 (6.36), 37–70	49.60 (6.57), 37–60	48.68 (6.21), 37–70
BDI-II, *mean (SD), range* ≥ 14, *n(%)*	4.22 (5.71), 0–32, 6 (8.7%)	4.85 (5.17), 0–19, 3 (11.5%)	3.84 (6.05), 0–32, 3 (7.0%)
RBDSQ, *mean (SD), range* ≥ *5, n(%)*	1.60 (1.67), 0–6, 4 (5.8%)	2.00 (1.53), 0–6, 1 (4.0%)	1.36 (1.72), 0–6, 3 (6.9%)
UPSIT total correct„ *mean (SD), range*	33.06 (5.27), 18–40, 21 (30.9%)	32.89 (5.23), 18–39, 11 (40.7%)	33.17 (5.37), 18–40, 10 (24.4%)
Hyposmia, *n (%)*			

*UPDRS-III, Unified Parkinson's Disease Rating Scale, motor section; MoCA, Montreal Cognitive Assessment; HVLT-R, Hopkins Verbal Learning Test—Revised; BDI-II, Beck Depression Inventory—II; STAI, State Trait Anxiety Inventory; REM Sleep Behavior Disorder Questionnaire; UPSIT, University of Pennsylvania Smell Identification Test*.

## Results

Thirty participants harbored a single *GBA* mutation, most with mild mutations (23 N409S, 1 R535H), two with a severe mutation (2 84GG), and four with other PD associated risk variants (2 T408M, 2 E362K), and 49 did not (controls). Among 30 *GBA*-NMC, 20 were recruited through a blood family member with PD, and 10 were spouses or friends. Seventeen *GBA*-NMC and 7 controls had a family history of PD in a first degree relative. Among the control group (non-*GBA*), all of whom were spouse (36) or friend (13) controls, 5 had family history of PD in first, and 2 had family history in first and in second degree relatives.

*GBA*-NMC (mean age ± SD: 53.93 ± 14.41 years, median 59.5, range 29–89) were not older than controls (59.90 ± 12.58, median 61.0, range 22–86). There were no group differences in sex (*GBA* mutation carriers 11 women; and controls 12 women) or years of education (*GBA* mutation carriers: mean ± SD 17.13 ± 1.78 years; controls: mean 16.78 ± 6.23 years). UPDRS-III, MoCA, HVLT-R Z-score, and Stroop interference *T*-score, and UPSIT scores did not differ between groups.

Table 1 shows results of linear regression models of hypothesized domains adjusting for demographics as appropriate. Stroop interference task was significantly associated with harboring a *GBA* mutation (*p* < 0.05), whereas UPDRS-III, HVLT-R, MoCA, UPSIT, presence, or significant symptoms of RBD or depression were not.

In the exploratory analyses, no difference was found between *GBA*-NMC and controls in the remaining cognitive measures listed above, or in anxiety, when correcting for multiple comparisons.

Forty percent of *GBA*-NMC were hyposmic, and 24% of controls were hyposmic. Among *GBA*-NMC, those with hyposmia had lower scores on the MoCA when controlling for age, (*p* = 0.018), with both hyposmia (*p* = 0.046) and age (*p* = 0.019) as significant predictors of MoCA score. Hyposmia was not significantly related to other primary outcome variables.

## Discussion

Findings from this study detail key associations between *GBA* mutation status and symptoms related to PD. *GBA* mutation carriers showed poorer performance on the inhibition component of the Stroop task. While there were no overall group differences in global cognitive functioning, assessed by the MoCA, *GBA* mutation carriers with hyposmia had lower global cognition scores than those without hyposmia. Additionally, *GBA* carriers were more likely to exhibit subtle motor signs.

Poorer performance in executive functioning tasks has been observed in *GBA* mutation carriers with and without parkinsonism (9, 28). We did not detect an association between *GBA* mutation status and global cognitive functioning, suggesting that overall there is limited, if any, deficit in most mild *GBA* mutation/variant carriers without PD. However, *GBA* mutation carriers did perform worse on the Stroop, an executive function task of response inhibition. Our study adds to the growing body of literature detailing a complex relationship between mutation status and performance on measures of global cognitive functioning (e.g., MoCA), albeit with a focus on mild mutation carriers. Most studies have reported worse performance on the MoCA among individuals with *GBA*-PD relative to idiopathic PD (1, 7), including a greater cognitive burden in severe mutation carriers (29). However, this may not occur early in disease as a recent large scale, multicenter study did not detect this difference among carriers, primarily of mild mutations (N409S), assessed early in the PD disease course (13). These prior studies suggest that among individuals with *GBA*-PD, disease duration, and mutation severity likely contribute to cognitive course.

In addition, *GBA* carriers without PD (*GBA*-NMC) have previously demonstrated greater cortical activation during a task of response inhibition (26), suggesting carriers may employ greater compensatory mechanisms in order to achieve similar performance, potentially revealing a mild burden of mutation status. Response inhibition, the ability to suppress a habitual or overlearned response, is a critical executive function, with cognitive and behavioral implications. Among *GBA*-NMC, such subtle cognitive changes may be indicative of an emerging disease process. Yet performance on this measure of response inhibition was within normal limits, reflecting mild, yet statistically significant difference between carriers and controls. Additionally, as relative weakness in this domain was mild, and as there were no differences between *GBA* mutation carriers and controls in other cognitive domains, this finding should be interpreted with caution.

Our finding of a subtle, isolated, relative cognitive weakness among *GBA*-NMC is in line with a prior study from our group that evaluated community dwelling older adults who were ascertained independent of mutation status. In that report, although global cognition was not worse, *GBA*-NMC did demonstrate greater, albeit mild, decline in verbal memory (24). Similar to the data reported herein, most participants had the mild *GBA* N409S mutation. These studies suggest that even among monoallelic carriers of mild mutations, relative cognitive vulnerabilities, which do not rise to the level of cognitive impairment, may be apparent. Our data do not disentangle the impact of mutation severity however, as the majority of the mutation carriers harbored mild mutations.

We did not detect an overall association between *GBA* mutation status and performance on a screening measure of global cognitive functioning (MoCA). This differs from results of a recent large, multicenter, study reporting worse performance on the MoCA among both *GBA*-NMC and *LRRK2*-NMC relative to control participants (22). Participants in this study were also predominantly mild mutation carriers. Our report also differs from a study of predominantly mid-life adult *GBA*-NMC comprised of both mild and severe mutation carriers, which found differences both at baseline and 2-year follow up on this measure (15). Of interest, 6-year follow up of this study, showed significant improvement in MoCA score among controls and improvement among carriers that did not reach the level of statistical significance (23). At follow up, while biallelic *GBA*-NMC performed significantly worse on the MoCA relative to controls, there was no longer a difference between monoallelic *GBA*-NMC and controls (23). Approximately half of the original *GBA*-NMC cohort was seen at 6 year follow up, raising the question of whether loss to follow up in that group was associated with worse cognition (23).

Further, our data support that while there was no difference between *GBA*-NMC and controls in overall olfaction scores, *GBA*-NMC carriers with hyposmia had poorer global cognitive functioning scores. This supports the longitudinal study showing olfaction as a potential preclinical marker, with reduced olfaction predicting worse cognition (23). While our relatively small sample size may have lacked the statistical power to detect an overall difference in MoCA score between carriers and controls, the relationship between olfaction and cognition in our sample may suggest variability in the impact of mutation status in our cohort of mild mutation carriers. Such a relationship emphasizes the need for continued, multi-modal assessment of this population in order to further our understanding of markers of disease burden, particularly among carriers of mild mutations.

Given that participants in this study were neurologically normal, there was limited variability in motor functioning scores, as neither *GBA*-NMC nor controls exhibited overt motor symptoms suggestive of PD. There were no group differences in continuous motor scores. As the UPDRS-III is a clinical tool, it may lack the sensitivity needed to determine if mild motor differences are present in our sample.

Our sample consisted predominantly of carriers of mild mutations (23/30 N409S, 1/30 R535H) or risk variants (2/30 T408M, 2/30 E362K). Penetrance of *GBA* mutations, particularly mild mutations and risk variants, is markedly reduced (21), and as such, most of the individuals in our sample are not expected to progress to PD. Other investigations which included a greater proportion of severe mutation carriers (15, 23) may yield evidence of greater disease burden that was not evident in our sample. Sensitivity analyses excluding the two carriers of more severe mutations (84 GG) yielded similar findings. Mutation carriers continued to show poorer performance on the inhibition component of the Stroop task when controlling for age, at the trend level, and carriers with hyposmia had lower scores on the MoCA when controlling for age.

Through our sample, we identified both *GBA*-NMC and control participants with and without family members with PD. Some of our sample was ascertained through affected family members, and others were spouses of individuals with idiopathic PD. Similar to other studies in which non-manifesting carriers and controls were recruited through tertiary care clinics, our participants with first degree relatives with PD, likely share additional risk factors that were not directly accounted for in this study, and a larger proportion of individuals with such a family history were in the *GBA*-NMC cohort. To determine the degree to which included mutation negative, but positive family history controls influenced the analysis, sensitivity analyses were performed, in which individuals in the control group with a family history of PD were excluded. These yielded a similar pattern yet did not reach statistical significance. Mutation carriers continued to show poorer performance on the inhibition component of the Stroop task when controlling for age, although this finding no longer reached statistical significance. In excluding these individuals, it is possible this analysis was limited by the reduced sample size. However, additional unmeasured genetic and environmental factors seen in family members of affected individuals interact with GBA status, and contribute to the differences measured.

Additionally, as limited genetic testing was available for this study, we are unable to conclusively determine if presence of additional genetic factors that may be associated with PD impact our findings. As additional genetic factors may modify *GBA* associated PD risk (30), along with the small sample size, incomplete genetic testing among both carrier and control participants limits degree of certainty that we are measuring solely the effect of *GBA* status.

This study reports subtle differences between *GBA*-NMC and mutation negative controls on non-motor features. While statistically significant differences emerged, it is notable that across domains assessed in this study, these differences were reflective of mild vulnerabilities. Motor functioning was within normal limits, and cognitive findings were limited to one domain, and reflective of mild, relative weakness, rather than significant burden. While such differences may be suggestive of an emerging pathological process, the overall disease burden was low in this sample. Our study was limited in that we were not able to disentangle the effect of mutation severity, as the sample of severe and risk variant carriers was low. Further, additional longitudinal investigations are needed to determine if these mild differences represent prodromal disease.

## Data Availability Statement

The raw data supporting the conclusions of this article will be made available by the authors, without undue reservation.

## Ethics Statement

The studies involving human participants were reviewed and approved by Mount Sinai Institutional Review Board. The patients/participants provided their written informed consent to participate in this study.

## Author Contributions

RS-P and EM: study concept and design. EM, RS-P, and MZ: drafting of the manuscript. EM, RS-P, CW, and RO: Statistical analysis. RS-P and MZ: study supervision. RS-P: obtained funding. All authors: acquisition, analysis, or interpretation of data, critical revision of the manuscript for important intellectual content, administrative, technical, or material support.

## Conflict of Interest

SB and RS-P have received consulting fees from Denali Therapeutics Inc. The remaining authors declare that the research was conducted in the absence of any commercial or financial relationships that could be construed as a potential conflict of interest.
